# India’s national maternity benefit cash transfer program and child anthropometry

**DOI:** 10.1038/s41598-026-48160-8

**Published:** 2026-04-18

**Authors:** Soumyajit Ray, Phuong Hong Nguyen, Sumantra Pal, Samuel Scott, Purnima Menon, Suman Chakrabarti

**Affiliations:** 1 Department of Agricultural, Environmental, and Development Economics, The Ohio State University, Columbus, OH, USA; 2 International Food Policy Research Institute, Washington, DC, USA; 3 Economic Adviser, Government of India, New Delhi, India

**Keywords:** Conditional cash transfer, Evaluation, Child health, Child growth, India, Health care, Medical research

## Abstract

This study evaluates the impact of India’s Pradhan Mantri Matru Vandana Yojana (PMMVY), the world’s largest rights-based conditional cash transfer (CCT) program targeting women during their first pregnancy. Using National Family Health Survey data from 2005 to 2021, we assess changes in growth in a nationally representative sample of children, before and after program implementation. To address potential biases, we employ a quasi-experimental approach with a Propensity Scores Matched Difference in Differences analysis. We find that potential exposure to the program is associated with moderate improvements (0.10 SD) in height-for-age and weight-for-age z-scores. These effects likely operate through increased uptake of health interventions and consumption of certain food groups. The study underscores persistent barriers to program access and finds that women from higher socio-economic strata have entered the CCT beneficiary pool since 2017, suggesting a need for more equitable program targeting.

**Supplementary Information:**

The online version contains supplementary material available at 10.1038/s41598-026-48160-8.

## Introduction

Low utilization of primary, preventive health care during pregnancy and early childhood is a key determinant of suboptimal maternal and child health outcomes, particularly in low- and middle-income countries (LMICs)^1^. Conditional Cash Transfer (CCT) programs incentivize low-income households to align their behaviour with national social objectives by directly providing cash when they meet specific conditions, such as ensuring children’s school attendance or receive immunizations^2^. Some cash transfers are designed to increase the demand for health interventions and enhance uptake of primary health care^3^. Currently, over 1.3 billion people in 100 countries have access to cash transfers^1,3–6^.

In various contexts, cash transfers have been associated with reductions in the risk of death among young children^6^, improved child health and nutrition outcomes among economically disadvantaged families^7^, and improved household diet quality^3^. Viewing cash transfers as strategic investments to enhance nutritional status during a child’s early years holds potential for long-term returns at individual and national levels. For India, investments made for a plausible set of nutritional interventions to reduce stunting are estimated to deliver a 34-fold return on investment in economic benefits^8^. The potential returns in India are particularly high given the large number of individuals who are positioned to benefit from such programs.

 In India, from 2005 to 2016, the Janani Suraksha Yojana (JSY) was the sole nationwide perinatal CCT program, providing coverage to over 10 million pregnant women^9,10^. However, in January 2017, the Indian Government introduced the Pradhan Mantri Matru Vandana Yojana (PMMVY) (which roughly translates to Prime Minister’s Maternity Benefit Program in English), another CCT program specifically catering to pregnant and lactating women in all districts throughout the country^11^. Every Indian pregnant and lactating woman is entitled to a maternity benefit of a minimum 6,000 Indian rupees (INR) under the National Food Security Act (NFSA) 2013. By capitalizing on the extensive reach and scope of the JSY, the PMMVY is potentially the world’s largest perinatal CCT program in terms of number of mothers reached^10^. In practice however, the eligibility of the PMMVY until 2022 was far from universal. While JSY money is conditional on institutional childbirths for the first two live births, offering a cash incentive ranging from INR 1,400 (rural) to INR 600 (urban) (Table S1), the PMMVY extended support of INR 5,000 (approximately US$ 58 in 2025) for only the first live birth upon fulfilling certain conditions. The sum of JSY and PMMVY fulfils the NFSA entitlement for eligible mothers. As on Jan 2020, PMMVY program had enrolled 54% of mothers of firstborn Indian children indicating less than universal coverage even among firstborns.

The PMMVY’s design draws inspiration from existing state specific CCT programs, namely the Muthu Lakshmi Scheme in Tamil Nadu and the Mamata Scheme in Odisha^12,13^. Under PMMVY, for their first pregnancy, women receive the first instalment of INR 1,000 upon completion of pregnancy registration within 150 days of pregnancy. The second instalment of INR 2,000 is after 180 days of pregnancy conditional and upon women completing at least one antenatal care check-up. The third instalment of INR 2,000 is paid upon completing childbirth registration and the first cycle of BCG, OPV, DPT and Hepatitis B vaccinations for the firstborn child.

PMMVY uptake may be suboptimal due to multiple implementation and behavioural barriers. These include conditionalities related to registration and documentation, requirements for bank accounts, state-specific supply-side constraints, limited awareness of entitlements, confusion with other maternal benefit schemes, mobility restrictions, and the relatively low value of the transfer. As a result, even among firstborns, the primary target group of the program until 2022, many eligible mothers were either excluded or chose not to participate. This population of eligible but non-beneficiary women thus provides a comparison group, albeit an imperfect one, for evaluating the program’s impact at scale.

Although prior studies have demonstrated the positive impact of state-specific schemes on enhancing the odds of receiving of essential health and nutrition interventions (counselling for breastfeeding, antenatal care, and vaccinations), improved food security outcomes, and reducing undernutrition (child stunting and anaemia), there is currently insufficient evidence regarding the effectiveness of the much larger PMMVY on these outcomes^12–14^. To address this evidence gap, we assessed the national impact of the PMMVY on children’s nutrition outcomes in India 5 years after implementation.

## Methods

### Data sources

We used mother-child and household-level data from three rounds of the Indian National Family Health Surveys (NFHS, equivalent to Demographic Health Surveys in other countries) in 2005-06 (NFHS-3), 2015–2016 (NFHS-4) and 2019–2021 (NFHS-5). These repeated cross-sectional surveys follow a systematic, multi-stage stratified sampling design, covering all states/union territories in India. While the 2005 round is representative at the national and state level, the 2015 and 2020 rounds are representative at the district level too. The 2005 round allows us to examine pre-intervention secular trends, the 2015 round serves as the pre-intervention baseline and the 2020 round provides post intervention period estimates, facilitating a pre-post comparison. We use data from only the first-born child for every mother in the sample because only these children were eligible for PMMVY. We exclude Tamil Nadu/Puducherry, Odisha, and Telangana from the analyses because they already or simultaneously implemented state-wide perinatal cash transfers^12,14–16^. After excluding children without valid anthropometric measurements, the final analysis sample was 96,250 children under five years old.

For coverage estimates we supplement NFHS data with cash disbursement data on PMMVY from the data bank of India’s parliament^17^ along with population data from the Health Management Information System and the Indian census.

### Disbursement and coverage of PMMVY

We estimate coverage of PMMVY in two ways. First, we use the total number of women who registered for PMMVY between 2017 and 2021. To convert absolute numbers to percentages, we use total live births in this period from Health Management Information System and the birth order distribution of Indian children from the Census to estimate an upper bound for PMMVY coverage. Second, the 2015 and 2020 NFHS rounds also provide data on women who received money from the JSY or any other perinatal cash transfer; along with the amount they received from JSY. It does not, however, ask any direct question about PMMVY. The proportion of mothers receiving INR 5,000 or more, is likely an underestimate of PMMVY coverage because women familiar with JSY would report accurate amounts (around INR 1,400 or less), whereas women unfamiliar with JSY may report higher amounts if they benefited from other transfers^18,19^.

### Outcomes

The primary outcomes are height-for-age z-score (HAZ), and weight-for-age z-score (WAZ)^20^. Child height and weight were measured by well-trained researchers using SECA-874U digital scales, SECA-213 stadiometers and SECA-417 infantometers^21^. We use age (in months), height (in centimetres), weight (in kg) and the WHO age-sex growth standards to calculate the z-scores for children aged 0 to 5 years^22,23^.

Secondary outcomes were program pathways including women (15–49 years) who registered pregnancy, women who received 4 or more antenatal care visits during pregnancy, women who consumed IFA for 180 + days during pregnancy, child (12–23 months) received all basic vaccinations, child (6–36 months) was given iron supplements, child (6–23 months) consumed dairy, flesh food, or fruits and vegetables.

### Covariates

Child-level covariates include age (months), sex (male/female). Mother-level covariates include height (cm), body mass index (kg/m^2^), age (years), and education (years)^24^. Household-level covariates include residence type (urban or rural), health insurance (binary), family size (number), religion (Hindu, Muslim, Christian), caste (disadvantaged, tribal) and wealth score. A wealth index was constructed with a principal component analysis of household’s characteristics including source of drinking water, type of toilet facilities, type of flooring, exterior wall material, type of roofing, cooking fuel, electricity, home ownership, domestic helper, number of household members per sleeping room, ownership of a bank or post office account and having a mattress, pressure cooker, chair, cot/bed, table, electric fan, radio/transistor, black-and-white television, color television, sewing machine, mobile phone, any other telephone, computer, refrigerator, watch or clock, bicycle, motorcycle or scooter, an animal-drawn cart, car, water pump, thresher, tractor and livestock (cows, camels, goats, horse, chicken, pigs)^25^. The index was constructed after pooling the NFHS rounds to obtain a consistent measure of asset poverty over time. In other words, within wealth quintiles, households have the same set of assets and amenities across NFHS rounds.

### Difference in differences estimating model

Random assignment of women to PMMVY would allow causal estimation of the average effect of the intervention. However, in the absence of a randomized assignment of individuals to treatment (i.e., exposure to PMMVY), we treated the timing and targeting of PMMVY as a natural (or quasi) experiment. For PMMVY, there may be unobservable child-specific factors (e.g., location, ingrained dietary habits, etc.) that are associated with receiving a cash benefit and child undernutrition. Such factors, however, are likely to be relatively invariant over the short-term. There may also be time-varying factors that could bias estimates, such as the national implementation of child health and nutrition programs. The preferred method of controlling for both issues is to use longitudinal data and estimate difference-in-differences (DID) models^26,27^. The DID estimation strategy requires that (1) outcome data be available before and after the intervention and (2) treatment and control (comparison) groups can be clearly distinguished. Here, examination of temporal changes within the treated group (between 2016 and 2021) controls for factors that don’t change in the short term. Accounting for change in outcomes within the comparison group acts as a counterfactual estimate for what would have happened in the absence of PMMVY and controls for factors that may change over time but are common to both groups. In other words, by looking at the changes in the treated group, while taking into account changes in the comparison group before and after the intervention, we can obtain the average treatment effect estimates^26^.

Finding an appropriate comparison group for PMMVY requires identifying children who did not receive PMMVY money, before and after PMMVY was implemented. NFHS provides variables for maternity benefit cash transfers including JSY, national or state governments. A combination of these provides an indicator comparing any perinatal CCT beneficiaries to non-beneficiaries^9,10^. After excluding states that have their own cash transfers and restricting the sample to first born children, we can reasonably isolate PMMVY from other transfers.

Yet, this comparison group is likely to suffer from selection bias stemming from the demand side; PMMVY non-beneficiaries may make health-specific demand choices due to unobserved factors that are systematically different from beneficiaries. To account for sources of bias we use Propensity Score Matching (PSM) along with DiD. First, we estimated a logistic regression model to predict the probability of receiving the treatment (cash transfer) based on observed covariates. Predicted probabilities from this model were then used to construct stabilized inverse probability weights: treated individuals were weighted by the inverse of their estimated probability of treatment, and untreated individuals by the inverse of one minus this probability. These weights were applied in subsequent outcome regressions, yielding estimates that account for differences in baseline characteristics and approximate a pseudo-population in which treatment is independent of observed confounders. In other words, using PSM improves exchangeability of participants in the sample, making them similar on observable variables that may confound results^28^. Exchangeability refers to the assumption that, after conditioning on observed variables, the treated and comparison groups have similar potential outcomes, enabling estimation of causal effects from observational data.

Our primary empirical strategy relies on pooling data from NFHS-4 and NFHS-5. To estimate the average treatment effect for PMMVY, we fit a DiD model using inverse probability weights with Eq. 1 for child *\:i*, born to mother *\:m*, from household *\:h,* in community *\:c,* in district *\:d,* in year of birth *\:t*:

1 $$Y_{{imhct}} = \beta _{0} + \beta _{1} CCT_{i} + \beta _{2} Post_{t} + \beta _{3} CCT_{i} * Post_{t} + D_{d} + \epsilon _{{imhcddt}}$$

2 $$Y_{{imhct}} = \beta _{0} + \beta _{1} CCT_{i} + \beta _{2} Post_{t} + \beta _{3} CCT_{i} * Post_{t} + \sum\limits_{{j = 4}}^{J} {\beta _{j} X_{{jimhcdt}} } + D_{d} + \epsilon _{{imhcdt}}$$

where $\:{Y}_{imhdt}$ is an outcome. $\:{CCT}_{i}$ is a treatment dummy that takes value 0 for non-CCT children, i.e. those born to mothers who reported that they did not receive any perinatal cash transfer, and 1 for CCT children. $\:{Post}_{t}$ is a time dummy that takes value 0 for data for children born before 2017 and 1 for children born after 2017. In Eq. 1, $\:{D}_{d}$ represents district fixed effects that account for time invariant district level factors and in Eq. 2, $\:{X}_{jimhdt}$ represents the vector of covariates child age, sex, survey wave and covid lockdown implemented during survey. $\:{\epsilon}_{imhdt}$ is an error term that represents residual variation. Standard error estimates are clustered at the state-level to account for intra-state correlations.

$\:{\beta\:}_{0}$ is the mean outcome for non-CCT firstborn children before 2017 (the reference group). $\:{\beta\:}_{1}$ is the difference in outcomes between CCT and non-CCT firstborn children before 2017. $\:{\beta\:}_{2}$ is the change in outcomes among non-CCT firstborns, before vs. after 2017. $\:{\beta\:}_{3}$ is the difference in difference estimator capturing the change in outcomes for CCT firstborns relative to non-CCT firstborns, before vs. after 2017. As the NFHS underreports exact transfer amounts and does not differentiate between JSY and PMMVY, our treatment variable reflects receipt of any perinatal cash transfer for first-born children. Consequently, the estimated difference-in-differences effects represent an intention-to-treat definition.

### Additional analyses

We conducted six additional analyses to strengthen the robustness of our findings. First, we estimate a continuous DiD model where the treatment intensity is defined as the cumulative per eligible beneficiary cash transfer amount disbursed by each state for each birth year since 2017. The variable used is calculated as the total amount disbursed divided by the total population of first-born children for each Indian state and each year since the program started. This continuous treatment variable, replaces the binary interaction term in Eq. 1, allowing us to estimate the dose-response relationship between the scale of cash received and child outcomes. By leveraging inter-state and inter-cohort variation in the magnitude of cash delivered, this approach accounts for heterogeneity in implementation intensity and provides policy-relevant insights on the marginal benefits of additional cash support. Second, we fit a model with Eq. 1, but we restrict it to the region of high common support where propensity scores range between 0.25 and 0.75. This helps ensure greater comparability among beneficiaries and non-beneficiaries. Third, we assess the sensitivity of our results to potential compositional changes within the CCT group following the introduction of PMMVY. Such changes may occur if implementation barriers lead to greater participation by more privileged mothers while excluding less privileged ones. To test this, we re-estimate Eq. (2) on sub-samples defined by fixed categories of maternal education (0–5 years, 6–10 years, 11–12 years, and ≥ 13 years) and household wealth (Q1 [poorest], Q2, Q3, and Q4–Q5 [wealthiest]). This approach ensures that comparisons are made within relatively homogeneous socioeconomic strata, thereby reducing the influence of changing group composition on estimated program effects. Fourth, we assess sensitivity to the age gradient in child growth by estimating effects within six-month age bins^29^. Fifth, a key identifying assumption of the DiD approach is that, in the absence of treatment, outcomes for the CCT group would have followed parallel trends to those of the non-CCT group. We test this assumption indirectly using NFHS-3 data by applying the propensity score model from the main specification to identify households that would likely have been CCT beneficiaries in 2005. We then plot trends in outcomes over time for CCT and non-CCT children to assess whether they moved in parallel prior to the intervention. Sixth, we use Eq. 1 to test whether PMMVY had an effect on intermediate variables that are known predictors of child anthropometry, in order to explore potential pathways of impact.

## Results

**Fig. 1:**
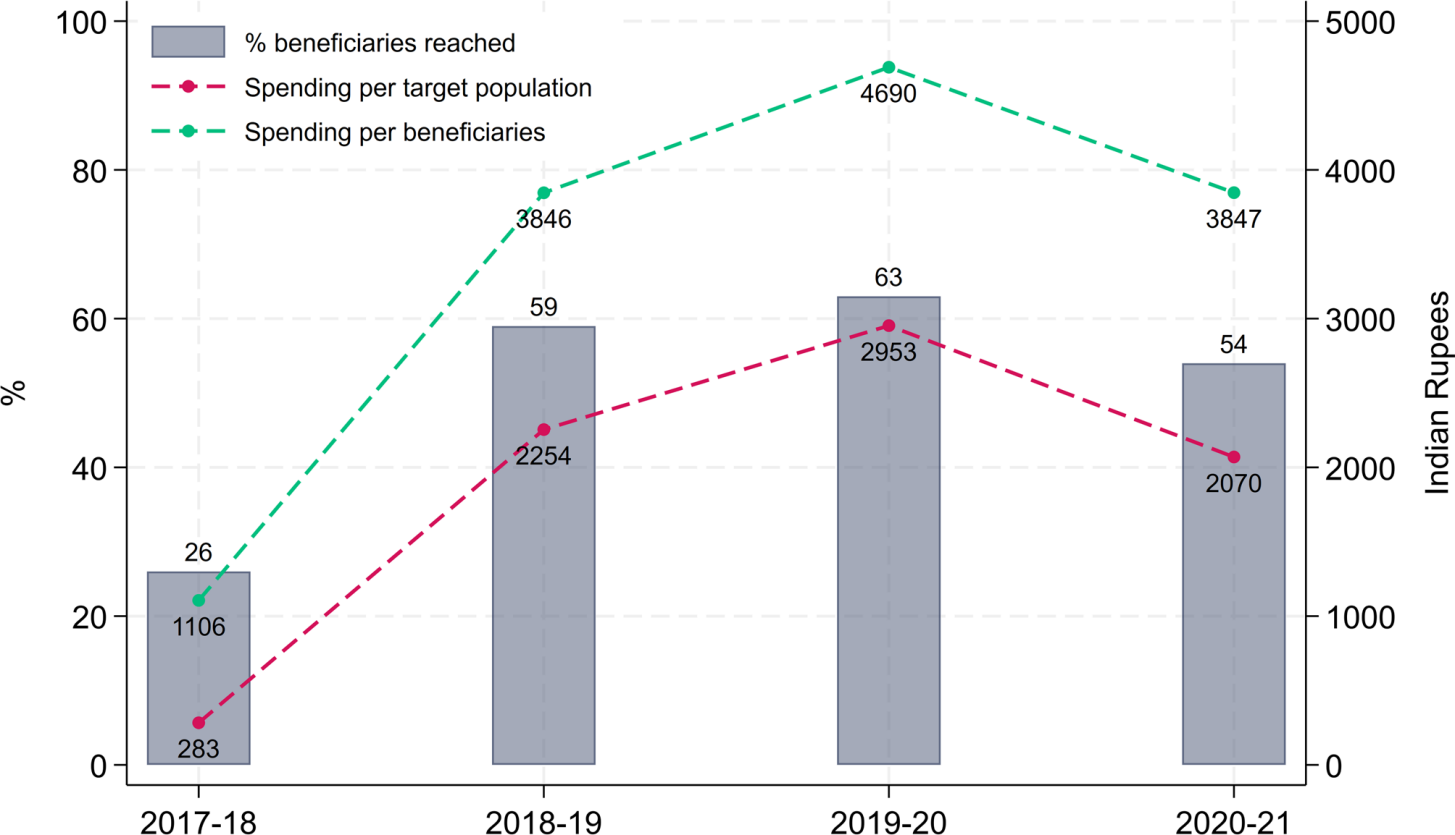
Resource allocation and program utilization for PMMVY between 2017 and 2021. Notes: PMMVY refers to Pradhan Mantri Matru Vandana Yojana. The coverage and expenditure figures are based on data from the health management information system, the census sample registration system, and responses filed by the ministry of women and child development in the Indian parliament.

Per Census and HMIS data, out of the 43.56 million firstborn children, 50.8% were covered by PMMVY between 2017 and 2020 on average. Figure 1 shows the trend in coverage of PMMVY since its rollout in 2017, with 63% of eligible beneficiaries being reached in 2019–20. However, the per-beneficiary disbursement as a proportion of total eligible beneficiaries was low, with the highest being INR 2,953 (approximately US$ 34 in 07/2025) per-beneficiary spending on the eligible population for 2019-20. Among beneficiaries who received PMMVY funds, disbursements ranged between INR 1,106 to INR 4,690, on average. Table 1 presents descriptive statistics comparing children born before and after 2017, stratified by whether the household reported receiving a maternity cash transfer. Among children born after 2017, 67% of households reported receiving INR 1,400 or less, while 33% received more than INR 1400 in benefits. In contrast, prior to 2017, 92% transfers were INR 1,400 or less, with very few reporting higher amounts.

**Table 1:** Summary statistics of outcomes and covariates among comparison groups among Indian first-born children aged 0–5 years.

	*Born before 2017*				*Born in or after 2017*			
	*Cash transfer*		*No cash transfer*		*Cash transfer*		*No cash transfer*	
	*Mean/%*	*SE*	*Mean/%*	*SE*	*Mean/%*	*SE*	*Mean/%*	*SE*
*Cash received in INR, %*								
< 600	5.0	(0.29)			0.8	(0.13)		
600–700	7.1	(0.27)			2.1	(0.16)		
701–1399	20.0	(0.45)			12.2	(0.47)		
1400	59.8	(0.50)			52.0	(0.57)		
1401–4999	6.9	(0.22)			14.3	(0.40)		
>=5000	1.1	(0.11)			18.7	(0.44)		
*Outcomes*								
Child length/height for age Z-score	−1.32	(0.02)	−0.98	(0.01)	−1.13	(0.02)	−0.93	(0.02)
Child weight for age Z-score	−1.52	(0.01)	−1.26	(0.01)	−1.29	(0.02)	−1.16	(0.01)
Mother registered pregnancy, %	93.0	(0.23)	89.8	(0.26)	97.4	(0.18)	94.2	(0.24)
Mother received 4 or more antenatal care, %	54.6	(0.48)	68.5	(0.40)	61.6	(0.54)	64.2	(0.47)
Mother consumed IFA for 180 + days, %	13.8	(0.39)	23.2	(0.40)	26.3	(0.51)	30.0	(0.47)
Child received all basic vaccinations, %	70.0	(0.77)	68.7	(0.84)	82.0	(0.67)	77.6	(0.68)
Child given iron supplements, %	24.6	(0.55)	29.3	(0.56)	39.6	(0.60)	37.1	(0.56)
Child consumed dairy, %	49.8	(0.77)	55.3	(0.74)	53.4	(0.75)	54.8	(0.69)
Child consumed flesh food, %	13.5	(0.60)	15.6	(0.63)	20.3	(0.67)	19.5	(0.60)
Child consumed fruits and vegetables, %	21.5	(0.71)	22.6	(0.63)	28.0	(0.69)	27.6	(0.65)
*Covariates*								
Urban residence, %	21.3	(0.46)	42.5	(0.45)	19.2	(0.50)	34.6	(0.52)
Health insurance, %	26.5	(0.44)	25.2	(0.38)	40.3	(0.55)	34.7	(0.46)
Household size, %	5.6	(0.03)	5.6	(0.02)	5.7	(0.03)	5.8	(0.03)
Hindu, %	81.1	(0.39)	78.2	(0.37)	82.3	(0.46)	79.7	(0.41)
Muslim, %	14.3	(0.37)	14.8	(0.32)	13.5	(0.43)	14.8	(0.37)
Christian, %	1.7	(0.10)	2.2	(0.12)	1.8	(0.17)	1.8	(0.12)
Scheduled caste, %	23.6	(0.44)	17.9	(0.36)	23.8	(0.48)	21.2	(0.41)
Scheduled tribe, %	12.6	(0.30)	6.6	(0.18)	11.2	(0.31)	8.7	(0.25)
Household wealth quintile	2.6	(0.01)	3.5	(0.01)	3.0	(0.01)	3.5	(0.01)
Mother’s height, cm	151.8	(0.06)	152.6	(0.05)	152.1	(0.07)	152.3	(0.06)
Mother’s body mass index, kg/m2	20.9	(0.04)	22.1	(0.04)	21.2	(0.04)	21.9	(0.04)
Mother’s age, years	23.8	(0.04)	24.9	(0.04)	23.4	(0.04)	24.0	(0.04)
Mother’s education, years	8.1	(0.05)	10.0	(0.04)	9.3	(0.05)	10.3	(0.04)
Child age, months	27.2	(0.16)	27.6	(0.15)	18.2	(0.11)	15.6	(0.10)
Male child, %	51.9	(0.49)	54.6	(0.44)	51.2	(0.57)	52.6	(0.50)
*Large Indian states*								
Bihar	10.5	(0.29)	6.1	(0.17)	8.7	(0.33)	9.4	(0.29)
Uttar Pradesh	15.5	(0.33)	11.4	(0.21)	22.7	(0.46)	17.4	(0.36)
Madhya Pradesh	10.9	(0.23)	3.9	(0.11)	10.9	(0.30)	4.5	(0.16)
Rajasthan	10.7	(0.25)	4.2	(0.12)	10.9	(0.32)	6.6	(0.21)
Maharashtra	4.0	(0.33)	16.1	(0.43)	5.1	(0.34)	12.1	(0.44)
Gujarat	2.3	(0.14)	8.2	(0.23)	3.3	(0.18)	6.3	(0.22)
Assam	8.3	(0.18)	2.3	(0.07)	3.9	(0.15)	2.7	(0.10)
Andhra Pradesh	2.2	(0.19)	4.6	(0.19)	4.0	(0.29)	3.2	(0.21)
Karnataka	4.2	(0.18)	7.1	(0.26)	2.3	(0.16)	6.6	(0.24)
Observations	21,556 (40%)		32,126 (60%)		13,719 (40%)		20,187 (60%)	

The denominators for food consumption variables are children 6–23 months, for all basic vaccinations are 12–23 months, and for child iron folic acid (IFA) are 6–36 months.

Among children born prior to 2017, those from households receiving cash transfers exhibited poorer anthropometric indicators, specifically lower height-for-age (HAZ) and weight-for-age (WAZ) Z-scores, relative to children in non-recipient households (Table 1). However, over time, both HAZ and WAZ improved at a faster rate among children in the cash transfer group. Improvements in maternal service use were also observed post-2017, with higher registration of pregnancy and uptake of antenatal care. However, coverage of IFA consumption for 180 + days remained low across all groups. Dietary indicators, such as consumption of dairy, flesh foods, and fruits and vegetables, showed modest improvements over time but were largely similar across transfer and non-transfer groups. Covariate distributions suggest modest differences in urban residence, wealth, and maternal education, with transfer recipients generally coming from more disadvantaged backgrounds.

**Fig. 2:**
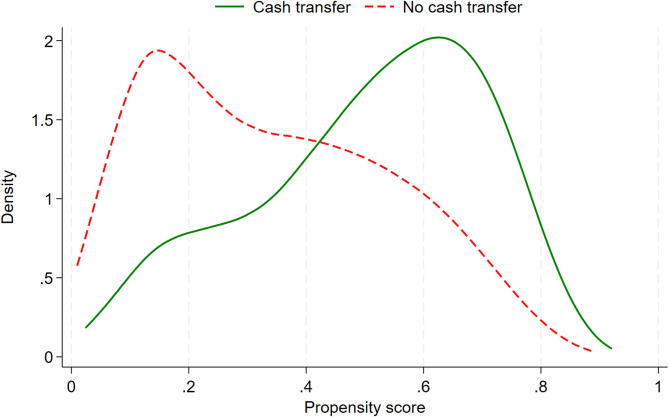
Evaluating common support for matching: propensity score overlap by treatment status. Notes: States with existing maternal benefits programs such as Tamil Nadu, Odisha, Telengana and Puducherry were dropped from the sample to isolate the PMMVY beneficiaries. Propensity scores calculated using state, health insurance, family size, Hindu, Muslim, Christian, scheduled caste, scheduled tribe, urban or rural status, socio-economic status score, mother’s height, mother’s age, mother’s education, child age, and child sex.

 Figure 2 illustrates the distribution of propensity scores for households that received a maternity cash transfer (green solid line) and those that did not (red dashed line). The overlap between the two distributions indicates the area of common support, where treated and comparison households have comparable probabilities of treatment, conditional on observed covariates. Overall, the visual inspection suggests that propensity score–based methods such as inverse probability weighting or matching can be applied to minimize selection bias. Comparison of Table 1 (before matching) and Table S3 (after matching) shows improved balance across treatment groups on key observable characteristics.

**Fig. 3:**
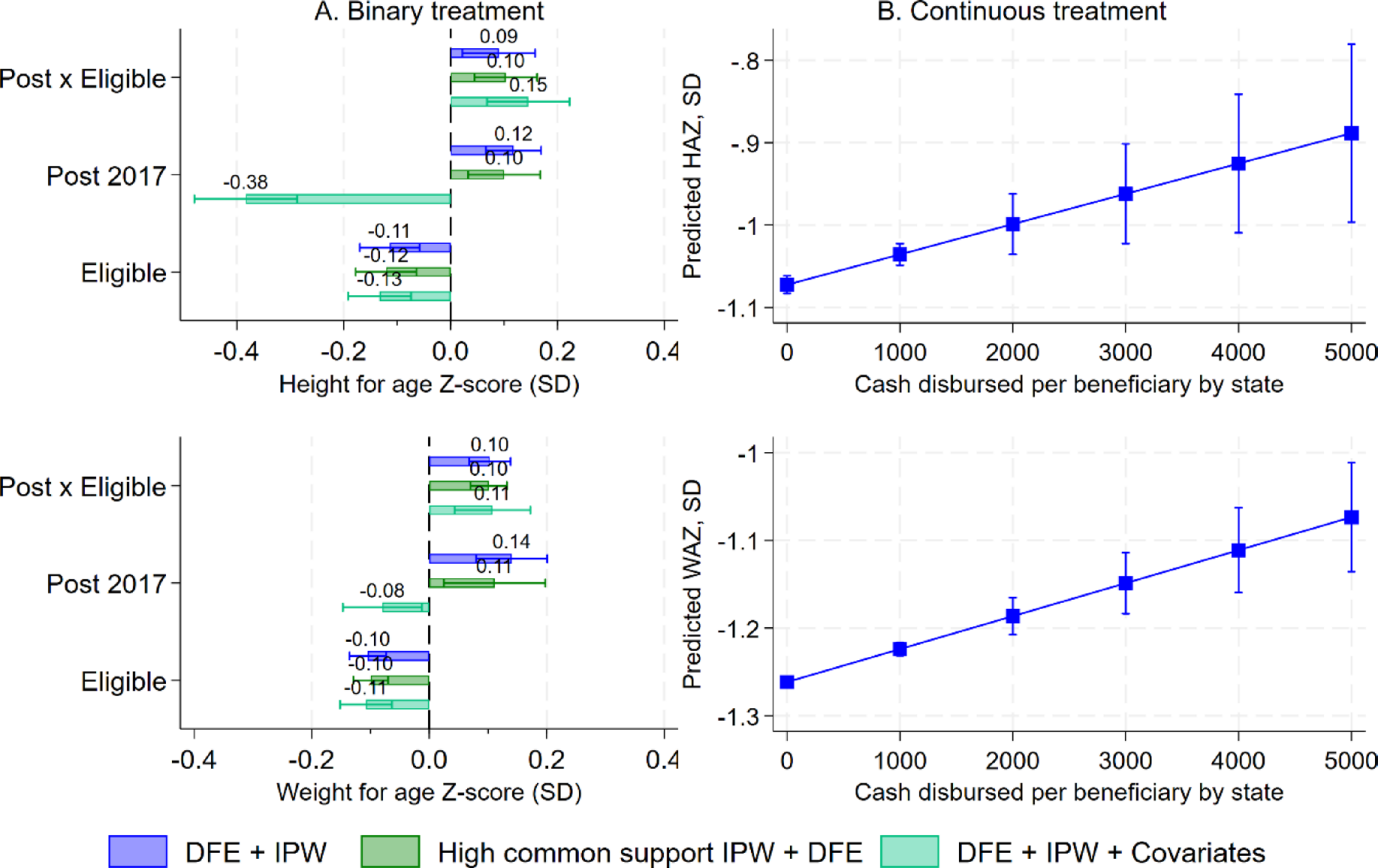
Propensity scores matched difference in difference ordinary least squares models for anthropometric outcomes among Indian firstborns 2015 and 2021. Notes: Post x cash is the difference in difference coefficient. All coefficients shown are propensity scores matched. Bands represent 95% confidence intervals. DFE + IPW is the district fixed effects and Inverse Probability Weights model. High common support restricts the DFE + IPW model to region of high common support (Propensity score > 0.25 and < 0.75). The DFE + IPW+Covariates model additionally controls for child age, sex, survey wave and COVID-19 lockdown. Propensity scores calculated using state, health insurance, family size, Hindu, Muslim, Christian, scheduled caste, scheduled tribe, urban or rural status, socio-economic status score, mother’s height, mother’s age, mother’s education, child age, child sex. Standard errors are robust and clustered at the state level. States with existing maternal benefits programs such as Tamil Nadu, Odisha, Telengana and Puducherry were dropped from the sample to isolate the PMMVY beneficiaries.

Figure 3 presents difference-in-differences estimates of the association between maternity cash transfers and child anthropometry. Panel A shows results using a binary indicator for whether a household was eligible to receive any cash, while Panel B shows margins plots of HAZ/WAZ across the total state-level cash amounts disbursed as a continuous treatment. The top panels report results for child HAZ and the bottom panels for WAZ. Across both specifications, the interaction term (DiD) between post-2017 birth and disbursement of cash transfer is positively associated with child nutritional outcomes. In panel A, three models are shown. The first includes district fixed effects and IPW, the second is run on a sub-set with high common support, and third includes all the covariates. Significant effects are observed for HAZ in all three models (β = 0.09 to 0.15 SD). Effects on WAZ are similar in magnitude (β = 0.10 to 11 SD). The continuous cash disbursed model also shows a positive association with HAZ and WAZ. The y-axis shows the range of predicted HAZ/WAZ differences across the range of cash disbursed (x-axis).

**Fig. 4:**
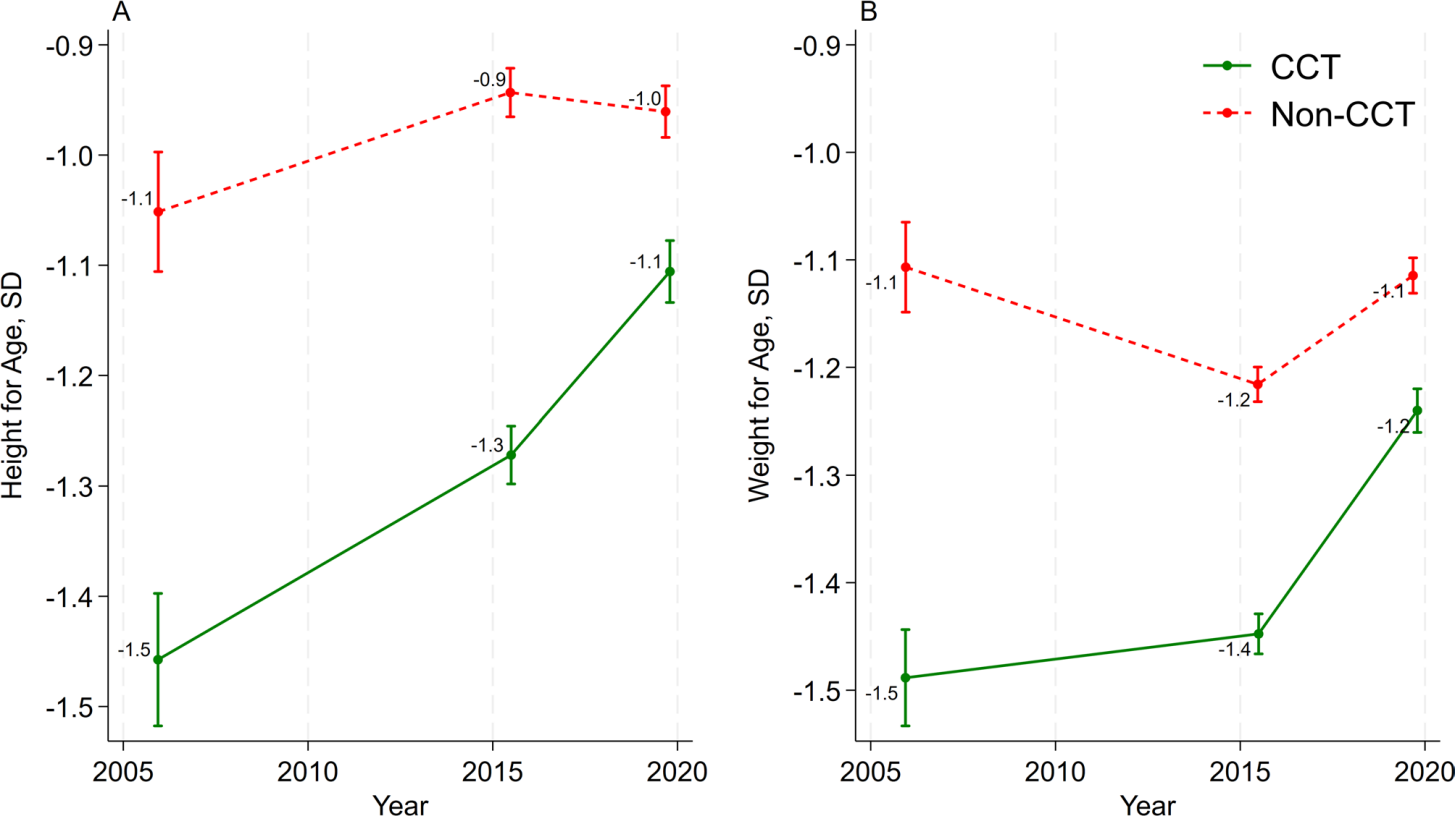
Pre intervention trends in outcomes by treatment status, 2005–2021. Notes: CCT=Condition cash transfer beneficiary. States with existing maternal benefits programs such as Tamil Nadu, Odisha, Telengana and Puducherry were dropped from the sample to isolate the PMMVY beneficiaries. Cash transfer beneficiaries in 2006 were predicted using state, health insurance, family size, Hindu, Muslim, Christian, scheduled caste, scheduled tribe, urban or rural status, socio-economic status score, mother’s height, mother’s age, mother’s education, child age, and child sex.

In Fig. 4, Panels A and B show HAZ and WAZ trends, respectively, for children in households predicted to be eligible for CCT (solid line) and those not eligible (dashed line). In the pre-intervention period (2005–2015), both groups exhibited similar trajectories, supporting the parallel trends assumption for HAZ. For WAZ, preintervention parallel trends do not appear to hold. After 2017, the CCT group experienced a noticeably steeper improvement in both HAZ and WAZ relative to the non-CCT group.

**Fig. 5:**
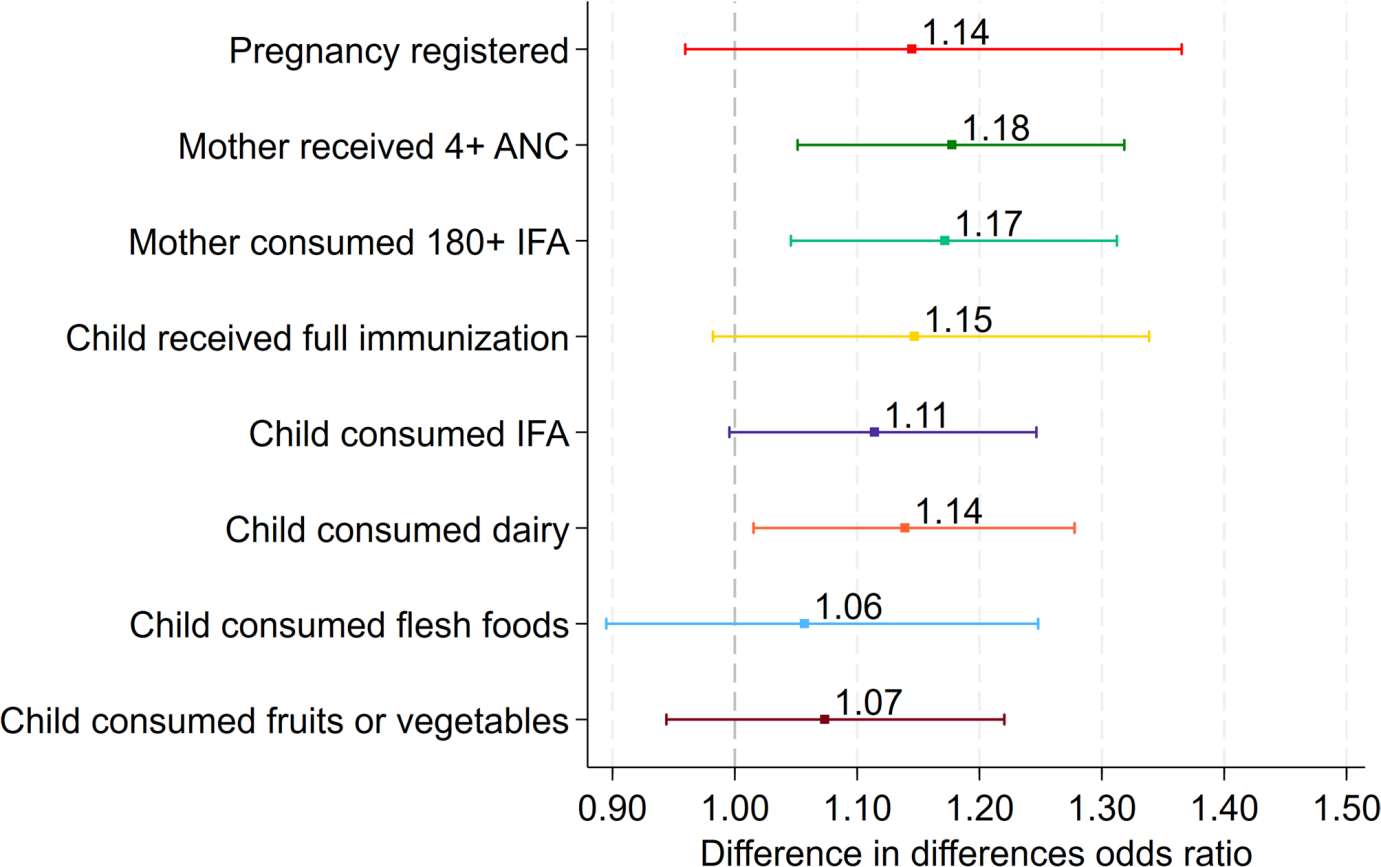
Propensity scores matched difference in difference logistic regression models for impact pathways among Indian firstborns 2015 and 2021. Notes: All coefficients shown are propensity scores matched difference in differences. All the outcome variables are in binary. Bands represent 95% confidence intervals. Models control for child age, sex, survey wave, COVID-19 lockdown and district fixed effects. Propensity scores calculated using state, health insurance, family size, Hindu, Muslim, Christian, scheduled caste, scheduled tribe, urban or rural status, socio-economic status score, mother’s height, mother’s age, mother’s education, child age, child sex. Standard errors are robust and clustered at the state level. States with existing maternal benefits programs such as Tamil Nadu, Odisha, Telengana and Puducherry were dropped from the sample to isolate the PMMVY beneficiaries.

Figure 5 shows improvements were observed among CCT mothers and children post 2017 in antenatal care (4 + ANC visits: OR = 1.18), maternal iron–folic acid (IFA) consumption (180 + days: OR = 1.17), and child immunization (OR = 1.15). Child dietary indicators also showed modest gains, with increased odds of dairy consumption (OR = 1.14), and IFA supplementation (OR = 1.11) among children.

**Fig. 6:**
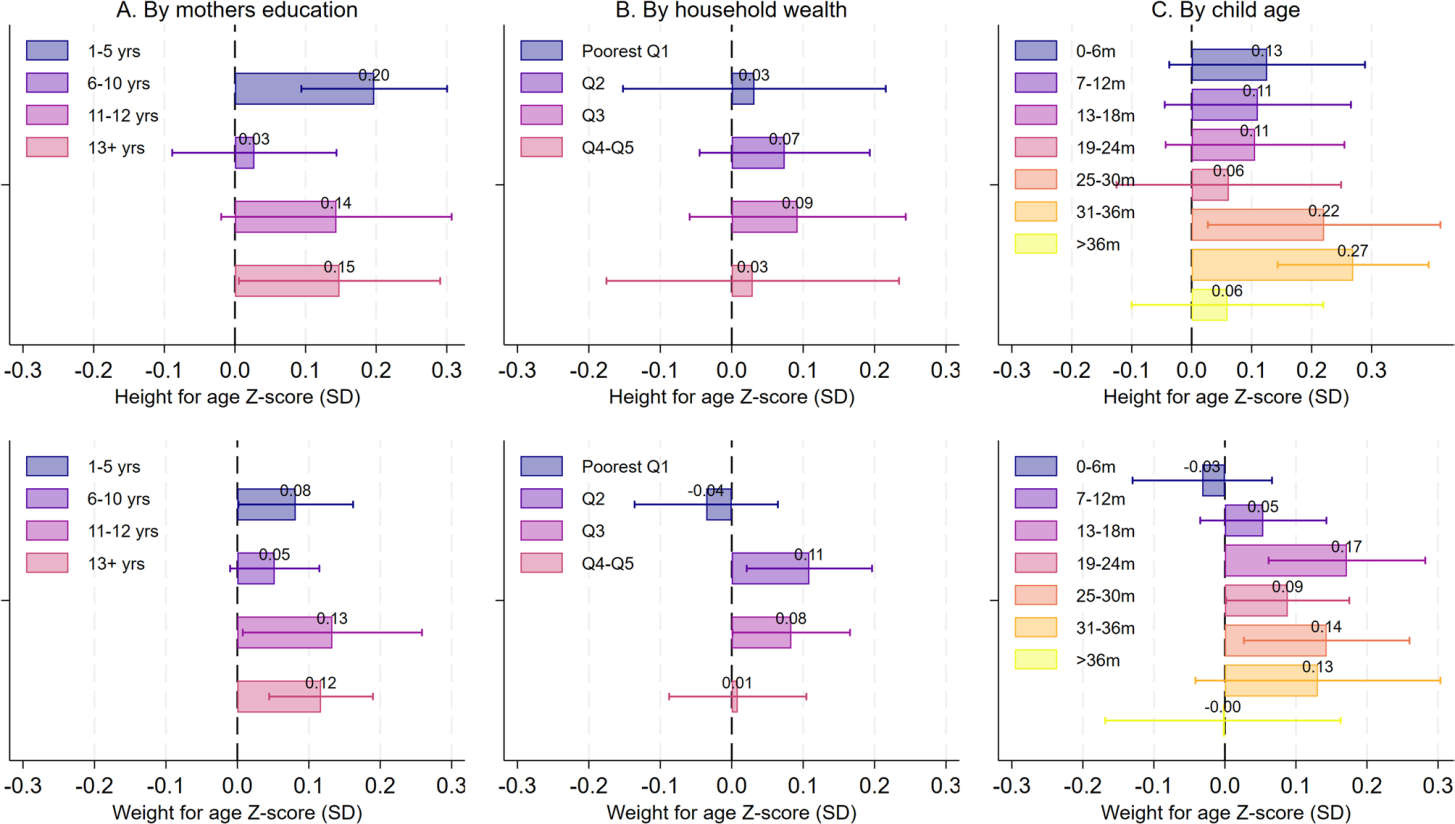
Propensity scores matched difference in difference stratified by categories of maternal education, household asset quintiles and child age 2015 and 2021. Notes: All coefficients are difference-in-difference and propensity scores matched. Bands represent 95% confidence intervals. Models control for child age, sex, survey wave and COVID-19 lockdown, district fixed effects. Propensity scores calculated using state, health insurance, family size, Hindu, Muslim, Christian, scheduled caste, scheduled tribe, urban or rural status, socio-economic status score, mother’s height, mother’s age, mother’s education, child age, child sex. Standard errors are robust and clustered at the state level. States with existing maternal benefits programs such as Tamil Nadu, Odisha, Telengana and Puducherry were dropped from the sample to isolate the PMMVY beneficiaries.

Figure 6 presents difference-in-differences estimates from Eq. (2), estimated separately for sub-samples defined by maternal education, household wealth categories and child age. Results by maternal education are broadly consistent in magnitude with those from the main specification. In contrast, estimates stratified by household wealth deviate from the primary results: effects on both HAZ and WAZ are attenuated, particularly among children from the poorest (Q1) and wealthiest (Q4–Q5) households. Notably, only the WAZ estimates remain statistically significant within these wealth-specific regressions. Lastly, age-stratified results show modest effects at 0–24 months, stronger effects at 24–36 months, and attenuation thereafter.

## Discussion

Our findings indicate that India’s PMMVY program is associated with moderate improvements in both service uptake and child growth outcomes. Using DiD models, we find that CCT beneficiary children born after the program’s scale-up in 2017 experienced significantly greater gains in height-for-age and weight-for-age compared to their non-CCT counterparts, with the largest improvements observed in states delivering larger transfer amounts per eligible beneficiary. Coverage of key maternal and child health interventions including maternal antenatal care, IFA consumption, and childhood immunization also improved more among CCT beneficiaries. Modest but positive changes were observed in child dietary practices, such as increased consumption of dairy. Taken together, these results suggest that well-targeted, nutrition-sensitive conditional cash transfers can strengthen uptake of essential interventions and contribute to improved child nutrition outcomes in resource-constrained settings.

Strengths of our study include the use of nationally representative, repeated cross-sectional survey datasets that establish temporal order, as exposure to the PMMVY precedes the studied outcomes. We employed DiD models with PSM that accounted for a comprehensive set of plausible confounders, thus supporting causal inference. To our knowledge, this is the first study that assesses the PMMVY program with respect to nutrition outcomes among children in India. Our results are relevant for LMICs where perinatal CCTs are being implemented.

Our study is not without limitations. First, survey datasets used in our study do not provide precise information on actual receipt of the PMMVY among women in India. Our analyses estimate population level average treatment effects that rely on aggregate trends^30^. Second, owing to the nonrandomized nature of PMMVY receipt, confounding from unobserved variables that correspond with both exposure to PMMVY and child nutrition outcomes cannot be ruled out. Given our DiD framework that comprehensively accounts for large potential confounders, any remaining biases could stem from unobserved factors that would (1) only affect CCT but not non-CCT children; (2) exhibit significant correlation with child anthropometry; and (3) not be completely accounted for by the control variables included in our analyses. Third, given the five-year gap between the two NFHS waves used in this study, maturation bias stemming from time-varying factors may likely bias our regression estimates^31^. Fourth, the estimated effects may be influenced by changes in the composition of the intervention group following the introduction of PMMVY. If PMMVY beneficiaries are relatively more privileged than JSY beneficiaries, the estimated impacts may partially reflect differences in socioeconomic advantage rather than the effect of the larger cash transfer alone. Accordingly, we caution readers against drawing strong causal inferences from these results and emphasize that the findings should be interpreted as indicative rather than definitive program effects.

The direction of coefficients on HAZ and WAZ for PMMVY estimated in the DiD model are in line with other studies evaluating maternal CCTs such as the JSY, IGMSY, or Mamta scheme in Odisha in the Indian context. For example, studies^32,33^ estimated that WAZ improved by 0.16 SD and 0.14 SD after the implementation of Mamata scheme. Similarly, whereas studies on Mamata and IGMSY reported modest and insignificant effects on HAZ, another study^34^ found 10% lower odds of stunting among CCT beneficiaries in Odisha. Moreover, global systematic reviews suggest that, on average, CCTs may improve HAZ by 0.024 SD, indicating a marginally higher impact of PMMVY for this outcome. Unlike most global CCTs which do not report significant impacts on WAZ, our models indicate that targeted CCTs can significantly improve this outcome.

A recent study commissioned in three states identified various hurdles that the beneficiaries face while availing the benefits of PMMVY^35^. It highlighted the procurement of documentation for enrolling into PMMVY as the primary hurdle^35^. To be eligible for these benefits, women must fill out lengthy documents for each of the three instalments. Aside from connecting their bank account with Aadhaar (the Government of India’s individual identification system), they must also show their “mother-child protection” (MCP) card, husband’s and their own Aadhaar card, and bank passbook^35^. Cooperation of Anganwadi workers and the child development project officers is also necessary for successfully filling the online application^36^. These hurdles make the procedure cumbersome, especially for women with minimal education^37^. Furthermore, banking-related issues such as inaccessibility of financial services, bank officials’ reluctance to provide zero balance accounts, and PAN card non-availability are also observed^35–37^. Even if beneficiaries are able to overcome these issues, women are far less likely than males to utilize and access their bank accounts and are thereby systematically excluded from the possible benefits of state-initiated transfers^38^.

Payment disbursement under PMMVY has often been delayed which restricts pregnant women’s ability to use the funds. It was also observed that the first instalment was often not provided to pregnant women in a timely manner. In some cases, beneficiaries got all three payments at once, after the childbirth^35^. This subsequently leads to beneficiaries spending the cash transfer amount on covering regular household expenditure rather than nutritious foods at critical points in first 1000 days^35^. PMMVY data on disbursements and beneficiary reach, suggests that implementation varies greatly across states. This was also observed for the JSY, which was implemented nationally. Further, limited publicly available data on PMMVY enrolments has been a major roadblock in studying the effect of COVID pandemic in its implementation. However, a study indicates that PMMVY enrolments declined from 73% in 2019-20 fell to 46% by 2021–22^39^.

Since its launch in 2017, the PMMVY has fallen short of reaching all entitled beneficiaries, largely due to persistent implementation barriers that limit equitable access. Despite these challenges, our findings indicate that receipt of maternity conditional cash transfers is associated with modest improvements in child anthropometric outcomes, potentially driven by increased utilization of maternal and child health services encouraged by program conditionalities. With the recent expansion of PMMVY eligibility to include second-born girls, the program has the potential to benefit millions more. Realizing this potential will require sustained efforts to reduce administrative burdens, strengthen outreach, and ensure that the program fulfills its mandate without exacerbating socioeconomic disparities in access.

## Supplementary Information

Below is the link to the electronic supplementary material.

Supplementary Material 1

## Data Availability

All datasets used in this study are publicly available and can be accessed through the National Family Health Survey (NFHS) portal and related government statistical repositories.
