# Supplementary material for: India’s national maternity benefit cash transfer program and child anthropometry
Source: Sci Rep. 2026 Apr 18;16:18807. doi: 10.1038/s41598-026-48160-8 (PMC13272589; doi:10.1038/s41598-026-48160-8)
Supplement: Supplementary file 1 — Supplementary Material 1 [file 41598_2026_48160_MOESM1_ESM.docx]

**Supplementary tables and figures**

**Table S1. Landscape of India’s perinatal conditional cash transfer programs, 1987-2021**

| **Scheme** | **Period** | **Region** | **Amount (INR)** | **Program Conditionality** | **Limits** |
| --- | --- | --- | --- | --- | --- |
| Muthu Lakshmi Reddy Maternal Benefit Scheme (MRMBS) | 1987 – present | Tamil Nadu – state | 18,000 splits into many tranches | Antenatal care (ANC), Iron Folic Acid (IFA) supplements, deworming, vaccinations | 2 live births |
| National Maternity Benefit Scheme (NMBS) | 1995 – 2005 | India – national | 500 | N/A | 2 live births  Woman should be from Below Poverty Line (BPL) cardholder family |
| Janani Suraksha Yojana (JSY) | 2005 – present | India – national | In Low Performing States (Uttar Pradesh, Uttaranchal, Bihar, Jharkhand, Madhya Pradesh, Chhattisgarh, Assam, Rajasthan, Odisha and Jammu and Kashmir): ₹1,400/- for rural areas and ₹1,000/- for urban areas.  In Other States (HPS): ₹700/- for rural areas and ₹600/- for urban areas. | Institutional delivery | Institutional delivery in government hospital, BPL card holder/ Scheduled Tribes (ST) / Scheduled Castes (SC) in private hospital |
| Mamata scheme (O) | 2011 – present | Odisha – state | 5,000 splits into 4 tranches | Pregnancy registered, ANC, IFA, Nutrition counseling, Vitamin A supplements, vaccinations | First 2 live births |
| Indira Gandhi Matritva Suraksha Yojana (IGMSY) | 2011 – 2016 | India – national^1^ | 4,000 splits into 3 tranches | Pregnancy registered, ANC, IFA, Nutrition counseling, first dose of child vaccinations (DPT, BCG, Polio) | First 2 live births |
| Pradhan Mantri Matru Vandana Yojana (PMMVY) | 2017 – present | India – national | 5,000 splits into 3 tranches^2^ | Pregnancy registered, ANC, first dose of child vaccinations (DPT, BCG, Polio) | First live birth^3^ |
| KCR Kit | 2017 – present | Telangana – state | 12,000 (Boy) 13,000 (Girl)  into 5 tranches | ANC, Institutional delivery, first and second dose of child vaccinations | First 2 live births, treatment in government hospital |
| Mamata scheme (G) | 2011 – present | Goa - state | 10,000 in 1 tranche | Institutional delivery | Live birth of girl child |

Note: Program details reflect the most recent identified for the scheme except in the case of PMMVY which is being evaluated in this paper. ^1^ Pilot program implemented in 52 out of the 640 districts in India. ^2^At the time of writing under the new program guidelines released in March 2022, the number of instalments under the scheme has been reduced from 3 to 2 instalments. ^3^As per the March 2022 guidelines, PMMVY benefits are being extended to a second child, but only if the second child is a girl. DPT stands for Diphtheria Pertussis & Tetanus vaccine and BCG stands for Bacillus Calmette Guerin single dose vaccine.

**Table S2. Instalment wise PMMVY layout**

| **Instalment** | **Conditionality** | **Documents Required** | **Amount (INR)** |
| --- | --- | --- | --- |
| First Instalment  (Registration) | • Register her pregnancy at any field functionary center along with required documents.  • Register her pregnancy within 150 days | • Application Form 1-A  • MCP Card  • Identity proof  • Bank/Post Office Account Passbook | 1,000 |
| Second Instalment | •At least one Ante Natal Care Check Up  • Can be claimed post 180 days of pregnancy | • Application Form 1-B  • MCP Card | 2,000 |
| Third Instalment | • Childbirth is registered  • Child has received first cycle of immunizations of BCG, OPV, DPT and Hepatitis B  • Aadhaar is mandatory in all states except for J&K, Assam, Meghalaya | • Application Form 1-C  • MCP Card  • Aadhaar ID  • Birth Certificate | 2,000 |

Source: (Sekher and Alagarajan, 2019)**;** DPT stands for Diphtheria Pertussis & Tetanus vaccine, BCG stands for Bacillus Calmette Guerin single dose vaccine and OPV refers to Oral polio vaccine. MCP card stands for Mother child protection card, while Aadhaar refers to unique identification cards provided by the government.

**Table S3. Summary statistics of covariates among comparison groups among Indian first-born children aged 0-5 years in the region of high common support**

|  | **Born before 2017** | | | | **Born in or after 2017** | | | |
| --- | --- | --- | --- | --- | --- | --- | --- | --- |
|  | Cash transfer | | No cash transfer | | Cash transfer | | No cash transfer | |
|  | Mean/% | SE | Mean/% | SE | Mean/% | SE | Mean/% | SE |
| **Covariates** |  |  |  |  |  |  |  |  |
| Urban residence, % | 21.5 | (0.33) | 22.1 | (0.28) | 14.8 | (0.37) | 13.0 | (0.29) |
| Health insurance, % | 26.0 | (0.34) | 26.1 | (0.32) | 43.1 | (0.49) | 44.5 | (0.46) |
| Household size, % | 5.7 | (0.02) | 5.7 | (0.02) | 5.7 | (0.03) | 5.7 | (0.02) |
| Hindu, % | 73.8 | (0.34) | 74.2 | (0.32) | 77.3 | (0.42) | 77.1 | (0.38) |
| Muslim, % | 13.9 | (0.26) | 14.0 | (0.26) | 13.6 | (0.34) | 13.1 | (0.31) |
| Christian, % | 7.6 | (0.20) | 7.2 | (0.19) | 5.6 | (0.23) | 6.3 | (0.22) |
| Scheduled caste, % | 19.0 | (0.30) | 19.0 | (0.29) | 21.6 | (0.41) | 22.2 | (0.39) |
| Scheduled tribe, % | 19.0 | (0.30) | 17.3 | (0.29) | 18.1 | (0.38) | 21.0 | (0.39) |
| Household wealth quintile | 2.8 | (0.01) | 2.9 | (0.01) | 3.0 | (0.01) | 2.8 | (0.01) |
| Mother’s height, cm | 152.1 | (0.04) | 152.2 | (0.04) | 152.2 | (0.06) | 151.8 | (0.06) |
| Mother’s body mass index, kg/m2 | 21.0 | (0.03) | 21.6 | (0.03) | 21.2 | (0.04) | 21.2 | (0.03) |
| Mother’s age, years | 24.3 | (0.03) | 24.5 | (0.03) | 23.7 | (0.04) | 23.4 | (0.03) |
| Mother’s education, years | 8.4 | (0.03) | 8.7 | (0.04) | 9.3 | (0.04) | 8.9 | (0.04) |
| Child age, months | 26.4 | (0.12) | 27.5 | (0.13) | 18.3 | (0.10) | 16.0 | (0.11) |
| Male child, % | 53.2 | (0.38) | 52.9 | (0.36) | 51.0 | (0.49) | 51.1 | (0.46) |
